# Novel Insights into the Management of Patients with Very High Cardiovascular Risk Eligible for PCSK9 Inhibitor Treatment: Baseline Findings from the PERI-DYS Study

**DOI:** 10.1007/s10557-022-07386-0

**Published:** 2022-09-30

**Authors:** Ulrich Laufs, Andreas L. Birkenfeld, Uwe Fraass, Bernd Hohenstein, Carsten Siegert, Jens Klotsche, Elisabeth Steinhagen-Thiessen, David Pittrow, Stefan Dexl, Sunnhild Salmen, Volker J. J. Schettler, Klaus G. Parhofer, Collaborators in the PERI-DYS Study, Sebastian Noé, Ulrike Spengler, Franz-Rudolf Fendler, Anselm Bäumer, Norbert Schön, Tilman Unger, Jens Taggeselle, Andreas Schwittay, Ulf Janseen, Frank Menzel, Christoph Axthelm, Andreas Wilke, Antje Spens, Ayham Al-Zoebi, Volker J.J. Schettler, Toralf Schwarz, Armin Jansen, Bernd Hohenstein, Alexander Stadelmann, Karin Eberhand, Katrin Borucki, Elisabeth Steinhagen-Thiessen, Axel Schlitt, Markus Knittel, Ann-Cathrin Koschker, Alexander Mann, Hans-Holger Ebert, Mohsen Tekiyeh, Katrin Gebauer, Ralf Spitthöver, Andrea Beigel, Ulrich Laufs, David Sinning, Norbert Jahnke, Beate Wild, Detlef Gysan, Johannes Ruef, Matthias Weißbrodt, Andreas Birkenfeld, Holger Killat, Steffen Bischoff, Johannes Beckermann, Ina Wittig, Jens Gerth, Peter Salbach, Dirk Raddatz, Wolfgang Ries, Markolf Hanefeld, Johannes Haas, Ilka Simon-Wager, Christian Zugck, Thomas Twisselmann, Volker Neumann, Markus Brode, Jens Ringel, Beate Schulze, Hansjörg Rothe, Wolfram Oettler, Alexander Stöckl, Sven Meyer, Sebastian Keßler, Rüdiger Meesters, Michael Brandt, Ksenija Stach-Jablonski, Berthold Amann

**Affiliations:** 1 Klinik und Poliklinik Für Kardiologie, Universitätsklinikum, Leipzig, Germany; 2 Innere Medizin IV – Diabetologie, Endokrinologie Und Nephrologie Am Universitätsklinikum, Tübingen, Germany; 3 Institut Für Diabetesforschung und Metabolische Erkankungen (IDM) des Helmholtz Zentrums München, Partner des Deutschen Zentrums Für Diabetesforschung (DZD E.V.), Munich, Germany; 4 Amgen GmbH, Muenchen, Germany; 5 Nephrologisches Zentrum Villingen-Schwenningen, Göttingen, Germany; 6 Deutsches Rheumaforschungszentrum, Berlin, Germany; 7 Department of Endocrinology and Metabolism, Charité Universitätsmedizin Berlin, Berlin, Germany; 8 Medizinische Fakultät, Technische Universität, Dresden, Germany; 9 Innovationszentrum Real-World Evidence, GWT-TUD GmbH, Dresden, Germany; 10 Nephrologisches Zentrum Göttingen GbR, Göttingen, Germany; 11 Medizinische Klinik und Poliklinik IV, Klinikum der Universität München, Munich, Germany

**Keywords:** Drug utilization, Secondary prevention, High risk, Statin intolerance, LDL cholesterol

## Abstract

**Aim:**

The PERI-DYS study aims to characterize two groups of patients with dyslipidaemia at very high CV risk: PCSK9i receivers and patients qualifying for but not receiving PCSK9i.

**Methods:**

This is an observational study by office-based and clinic-based physicians, mainly cardiologists and other internists in Germany, with data extracted from patient charts. ClinicalTrials.gov identifier NCT03110432.

**Results:**

A total of 1659 patients were enrolled across 70 sites. The majority of patients (91.0%) were reported as having mixed dyslipidaemia or non-familial or heterozygous familial hypercholesterolemia. At enrolment, 794 (47.9%) of patients were PCSK9i receivers (of these 65.9% ongoing, and 34.1% newly treated within 30 days before their baseline visit). Among PCSK9i receivers, the majority had evolocumab 140 mg (*n* = 632, 38.1% of total). PCSK9i receivers compared to non-receivers were about 2 years younger and had a lower proportion of males. In terms of comorbidities, they had (statistically significantly) more often CAD, and less often PAD, diabetes mellitus, arterial hypertension and chronic renal disease. The calculated untreated median LDL-C was 187 mg/dl (IQR 127; 270) in ongoing PCSK9i receivers, 212 mg/dl (IQR 132; 277) in newly treated PCSK9i receivers, and 179 mg/dl (IQR 129; 257) in non-receivers. Physician-reported statin intolerance was much more common in the two PCSK9i receiver groups as compared to non-receivers (67.3% versus 15.3%). Consequently, patients in the PCSK9i groups received fewer concomitant statins. Mean total cholesterol (143 vs. 172 mg/dl) and LDL-C (69 vs. 99 mg/dl) were considerably lower in ongoing PCSK9i receivers compared to non-receivers.

**Conclusions:**

PCSK9i receivers are characterized by higher baseline LDL-C and a higher portion of statin intolerance compared to those qualified for but not-receiving PCSK9i treatment. On-treatment, LDL-C was lower in PCSK9i receivers. Ongoing follow-up will determine the prognostic importance of these findings.

**Supplementary Information:**

The online version contains supplementary material available at 10.1007/s10557-022-07386-0.

## Introduction

Based on solid evidence from clinical trials, LDL-C lowering is recommended for the prevention of cardiovascular (CV) outcomes [1–3]. The EAS/ESC European Dyslipidaemia guidelines 2019 recommend a treatment goal of LDL-C < 55 mg/dl (1.4 mmol/L) and a > 50% LDL-C reduction for patients with very high risk [4], whereas the former treatment goal of EAS/ESC 2016 guidelines was < 70 mg/dl (1.8 mmol/L) or 50% reduction of LDL-C [4]. In addition to lifestyle changes and therapy with statins and ezetimibe, the current guidelines recommend PCSK9 inhibitors (PCSK9i) especially in patients with very high risk and LDL-C far from goal [5]. Two fully human monoclonal antibodies against the proprotein convertase subtilisin kexin like type9 (PCSK9) protein inhibitors, evolocumab and alirocumab, are available since 2015 [6]. These PCSK9i lower LDL-C by an additional 50–60% on top of oral lipid lowering therapies and are very well tolerated. Large outcome trials have demonstrated the additional reduction of CV risk under PCSK9i in well-treated patients in secondary prevention [7, 8].

Despite this evidence, access to PCSK9i in many countries is limited by national/local regulations. In Germany, reimbursement of PCSK9i by statutory health insurance is regulated by the Federal Joint Committee (G-BA), the highest decision-making body of the joint self-government of physicians, hospitals, and health insurance [9, 10]. The G-BA regulation defined a narrow patient population (within the CV very high-risk patient population) that are eligible for reimbursement without the need of application prior to initiation of treatment. One key criterion to define this patient group is lack of LDL-C treatment target fulfillment despite maximally tolerated lipid lowering therapy [11]. Further criteria include documented treatment with statins for 12 months in principle, LDL-C above goal despite optimal oral therapy, and initiation of PCSK9i by a specific board-certified specialist only.

However, despite these regulations, significantly more patients qualify for PCSK9i than the actual number of prescriptions indicate. The reasons why some patients receive and others do not receive PCSK9i are incompletely understood. Therefore, the PERI-DYS study aims to describe and compare two groups of patients with dyslipidaemia at very high CV risk: those treated with PCSK9i compared with patients qualifying for but not treated with PCSK9i.

## Patients and Methods

### Ethical Considerations

The study protocol was approved by the institutional review board of the Medical Faculty of the Technical University of Dresden, Germany (EK 4,761,120,166) and all patients provided written informed consent. The study was registered by the regulatory authority Paul Ehrlich Institut under NIS384 and by ClinicalTrials.gov under NCT03110432. Responsible party (legal sponsor) is GWT-TUD GmbH.

#### Design and Setting

PERI-DYS is a prospective, multi-center, observational registry study with a 3-year follow-up period. Hospital- and office-based physicians who regularly treated patients with dyslipidaemia, mostly internists with a specialization in cardiology, nephrology, or diabetology and endocrinology (including lipidology) were invited to participate. Cardiologists were the largest investigator group.

#### Study Cohort and Data Collection

Patient inclusion criteria were (1) familial, homozygous hypercholesterolemia, in whom pharmaceutical and diet options for lipid lowering have proved insufficient, or (2) confirmed familial, heterozygous hypercholesterolemia under consideration of the total familial risk, or (3) heterozygous familial or non-familial hypercholesterolemia or mixed dyslipidaemia with therapy refractory course; maximal dietary and pharmaceutical lipid lowering therapy—documented for basically a 12-month period; with unsatisfactorily lowered LDL-C value (and thus eligible for LDL apheresis); confirmed vascular disease; other risk factors for cardiovascular events. Further, patients had to be at least 18 years old, and suitable for follow-up in a long-term study with regard to adherence, comorbidities and prognosis. The only exclusion criterion was the concurrent participation of the patient in a randomized clinical trial. The sites were requested to document a balanced share of patients on patients on PCSK9i and those eligible for but not receiving PCSK9 antibodies (to avoid overrepresentation of the former group).

All diagnostic and therapeutic decisions were on discretion of the treating physician. Commercially available drugs had to be used. However, dosing of PCSK9i, if used, had to adhere to the stipulations made in the package insert (posology). Further, treatment with one of the two registered PCSK9i antibodies had to be in line with the criteria set forth in the two final versions of the “prescription restriction” documents (Arzneimittelrichtlinie Repatha® or Praluent®) [9, 10]. Patients could switch between PCSK9i brands, i.e., from evolocumab to alirocumab and vice versa, or from/to other lipid lowering drugs.

A web-based electronic CRF was used for data recording. All data was entered by designated staff of the participating sites. The list of parameters to be documented (if available) at inclusion or during follow-up is shown in Supplementary Table 1.

#### Outcome Measures

The following outcomes were defined in the protocol:

- *Primary:* Describe patient and disease characteristics and LDL-C goal achievement (for patients at very high risk < 70 mg/dl, < 1.8 mmol/L) in patients qualifying for PCSK9i use under G-BA regulations, especially comparing PCSK9i receivers vs. non-receivers under real-life circumstances in Germany.
- *Secondary:* Describe patient and disease characteristics and LDL-C goal achievement (< 70 mg/dl, < 1.8 mmol/L) at office-based cardiologists vs. specialized lipid ambulances in comparison of the two subgroups PCSK9i receivers vs. non-receivers.

#### Statistical Analysis

Categorical variables were reported in frequency tables including information on absolute and relative frequencies as well as the number of missing values. Continuously distributed variables were analyzed by reporting the sample median and interquartile range. Due to the distribution pattern, the medians of the calculated LDL-C values and other lipid parameters are presented in this paper.

The current analysis represents a cross-sectional dataset at inclusion into the registry. Data are presented for the total cohort, and separately for PCKS9i receivers or non-receivers at baseline. If the duration of PCSK9i therapy likely influenced lipid laboratory parameters (mainly LDL cholesterol), we made a further differentiation between patients who were receiving PCSK9i at baseline for at least 30 days (pre-treated) or were newly treated with PCSK9i.

All values are presented as they were reported by the sites.

The purpose of “calculation of lipid values prior to any lipid-lowering therapy in the patient’s past,” for each patient on ongoing lipid lowering treatment, the initial LDL-C values were calculated under the assumption for an average LDL-C reduction according to the EAS/ESC 2019 Dyslipidaemia Guidelines [5]. In patients without current PCSK9i treatment (including those with PCSK9i treatment initiation < 1 week before baseline, as an effect could not yet be expected), the following LDL-C reduction was assumed: low intensity statin 20%, moderate intensity statin (and statins with unknown intensity or unknown dose) 30%, high intensity statin 50%, high intensity statin plus ezetimibe 65%. In patients on ongoing PCSK9i therapy (1 week or longer), the following LDL-C reduction was assumed: PCSK9i alone or PCSK9i plus low or moderate intensity statin 60%, PCSK9i plus high intensity statin 75%, PCSK9i plus high intensity statin plus ezetimibe 85%, respectively. Statin or ezetimibe were considered in the calculations if they were ongoing for at least 5 days at baseline.

Lipid-lowering therapies were only considered in the calculation model if they were ongoing for 5 days or longer at baseline (no discontinued medication).

Information on concomitant diseases was collected using tick boxes for the most relevant diseases and free text fields. Similarly, information on lipid-lowering medications and cardiovascular medications was collected. Medication was coded with WHO-DD Drugs Insights by ATC codes. Statistical analyses were conducted with the software package SAS version 9.3 (SAS Institute Inc, Cary, NC, USA) or higher.

## Results

### Demographics and Comorbidities of the Total Cohort

  At database cut-off (05 July 2021), a total of 1659 patients were included in the database. Demographic characteristics and comorbidities at inclusion (baseline) are shown in Table 1. Median age was 63 years, two-thirds of patients were males (65.4%). Median BMI was 28 kg/m^2^, and 30.8% were obese (BMI ≥ 30 kg/m^2^). All patients were at very high cardiovascular risk by their treating physicians. The great majority of patients had statutory health insurance (94.5%).

**Table 1:** Demographics, risk factors, FH type, and comorbidities at baseline

Parameter	Total *N* = 1659	PCSK9i receiver *N* = 794	PCSK9i non-receiver *N* = 865	*P* value
General				
Female	574 (34.6%)	303 (38.2%)	271 (31.3%)	0.003
Male	1085 (65.4%)	491 (61.8%)	594 (68.7%)	
Age (years), median (IQR)	63 (56; 71)	62 (56; 70)	64 (56; 73)	0.003
BMI (kg/m^2^), median (IQR)	27.5 (25.0; 31.0)	27.8 (25.0; 31.2)	27.4 (24.9; 30.8)	0.239
Homozygous FH	4 (0.2%)	4 (0.5%)	0 (0.0%)	0.038
Heterozygous FH	145 (8.8%)	74 (9.3%)	71 (8.2%)	0.478
Combined/mixed hyperlipidemia	1,510 (91.0%)	716 (90.2%)	794 (91.8%)	0.290
BP systolic (mmHg), median (IQR)	133 (121; 144)	134 (121; 145)	132 (121; 142)	0.114
BP diastolic (mmHg), median (IQR)	80 (72; 85)	80 (72; 85)	80 (73; 85)	0.748
Comorbidities				
CAD	1161 (71.2%)	591 (74.4%)	570 (68.2%)	0.015
CVD	194 (11.9%)	104 (13.1%)	90 (10.8%)	0.346
PAD	298 (18.4%)	103 (13.1%)	195 (23.5%)	< 0.001
Combination of CAD plus CVD and/or PAD	247 (15.4%)	114 (14.7%)	133 (15.9%)	0.561
Diabetes mellitus	438 (26.9%)	186 (23.5%)	252 (30.2%)	0.008
Type I Diabetes mellitus	20 (1.2%)	10 (1.3%)	10 (1.2%)	0.847
Type II Diabetes mellitus	409 (24.7%)	174 (21.9%)	235 (27.2%)	0.013
Arterial hypertension	1,324 (81.3%)	627 (79.0%)	697 (83.6%)	0.045
Chronic kidney disease^a^	185 (11.4%)	87 (11.0%)	98 (11.8%)	0.037
Socio-economic				
Insurance				
Public	1502 (94.5%)	737 (95.3%)	765 (93.6%)	0.137
Private	88 (5.5%)	36 (4.7%)	52 (6.4%)	

*CAD* coronary artery disease, *CVD* cerebrovascular disease, *ASCVD* atherosclerotic cardiovascular disease

^a^eGFR < 60 ml/min/1.73 m^2^

Four patients had homozygous FH (0.2%), 145 heterozygous FH (8.8%), and 1510 (91.0%) combined/mixed dyslipidaemia. Comorbidities were frequent, in particular arterial hypertension in 81.3%, coronary arterial disease in 71.2%, cerebrovascular disease in 11.9%, peripheral arterial disease in 18.4%, and diabetes mellitus in 25.9%. Chronic kidney disease was reported in 11.4%.

#### Lipid-Lowering and Cardiovascular Medication at Baseline Visit

Overall, there were 794 PCSK9i receivers at inclusion (of these, 65.9% on ongoing therapy and 34.1% newly treated). Then, 88.7% had evolocumab and 11.3% had alirocumab.

Further, there were 865 patients eligible for PCSK9i, but non-receivers at baseline (52.1%). Of the latter, most were on statins (51.5% only statin, 37.5% statins combined with other LLT).

**Fig. 1:**
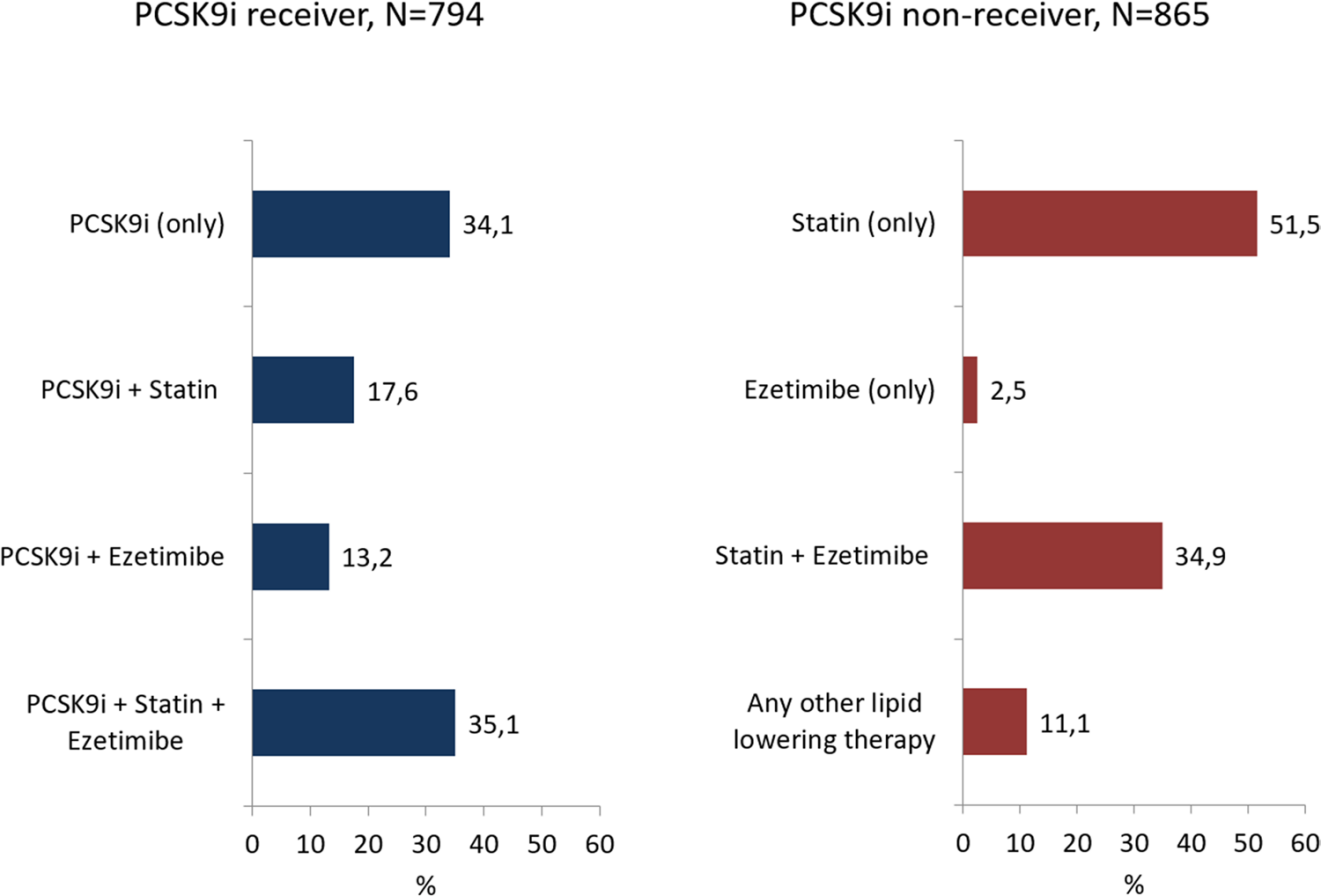
Lipid lowering therapy at inclusion, by group

Details about LLT are provided in Table 2 and Fig. 1. PCSK9i only were given to 16.3% of the total population, in combination with statins in 8.4%, in combination with ezetimibe in 6.3%, in combination with both statins and ezetimibe in 16.8%. Conversely, statins alone were administered in 26.9%, in combination with ezetimibe in 18.2%. Ezetimibe alone was given in 1.3%, therapy other than the described groups in 5.8%. Overall, nicotinic acid, fibrates, cholestagel, and omega-3 fatty acids were rarely used (data not shown).

**Table 2:** Lipid-lowering medication at baseline visit

Parameter	Total *N* = 1659	PCSK9i receiver *N* = 794	PCSK9i non-receiver *N* = 865	*P* value
Evolocumab 140 mg	632 (38.1%)	632 (79.6%)		
Evolocumab 420 mg	72 (4.3%)	72 (9.1%)		
Alirocumab 75 mg	86 (5.2%)	86 (10.8%)		
Alirocumab 150 mg	4 (0.2%)	4 (0.5%)		
PCSK9i (only)	271 (16.3%)	271 (34.1%)		
PCSK9i + Statin	140 (8.4%)	140 (17.6%)		
PCSK9i + ezetimibe	105 (6.3%)	105 (13.2%)		
PCSK9i + Statin + ezetimibe	278 (16.8%)	278 (35.1%)		
Statin (only)	445 (26.9%)		445 (51.5%)	
Ezetimibe (only)	22 (1.3%)		22 (2.5%)	
Statin + ezetimibe	302 (18.2%)		302 (34.9%)	
Any other lipid lowering therapy	96 (5.8%)		96 (11.1%)	
Any statins at baseline	1,165 (70.2%)	418 (52.6%)	747 (86.4%)	< 0.001
Low intensity*	46 (4.0%)	27 (6.5%)	19 (2.5%)	0.004
Moderate intensity**	498 (42.8%)	174 (41.6%)	324 (43.4%)	
High intensity***	621 (53.2%)	217 (51.9%)	404 (54.1%)	
on ezetimibe at baseline	707 (42.6%)	383 (48.2%)	324 (37.5%)	< 0.001
on fibrate at baseline	22 (1.3%)	12 (1.5%)	10 (1.2%)	0.527
on lipid apheresis at baseline	51 (3.1%)	29 (3.7%)	22 (2.5%)	0.191

^*^Low intensity: Fluvastatin 20 to 40 mg; Lovastatin 20 mg; Pitavastatin 1 mg; Pravastatin 10 to 20 mg; Simvastatin 10 mg

^**^Moderate intensity: Atorvastatin 10 to 20 mg; Fluvastatin 40 mg 2 × /day, 80 mg; Lovastatin 40 mg; Pitavastatin 2 to 4 mg; Pravastatin 40 to 80 mg; Rosuvastatin 5 to 10 mg; Simvastatin 20 to 40 mg

^***^High intensity: Atorvastatin 40 to 80 mg; Rosuvastatin 20 to 40 mg

Any cardiac comedication was given in 88.2% (1 in 15.4%, 2 in 33.2%, 3 in 30.0%, 4 or more in 9.6%) (Table 3). The most frequently prescribed groups were antiplatelets (ASS in 69.3%, clopidogrel in 11.7%), beta blockers (60.8%), ACE inhibitors (35.0%), and calcium antagonists in 21.4%. Insulin was given in 7.8%, oral antidiabetic drugs in 15.7%.

**Table 3:** Cardiovascular medication at baseline

Parameter	Total *N* = 1659	PCSK9i receiver *N* = 794	PCSK9i non-receiver *N* = 865	*P* value
Acetylic salicylic acid	1,150 (69.3%)	557 (70.2%)	593 (68.6%)	0.481
Clopidogrel	194 (11.7%)	79 (10.0%)	115 (13.3%)	0.034
NOAC	212 (12.8%)	82 (10.3%)	130 (15.0%)	0.004
Dabigatran	6 (0.4%)	3 (0.4%)	3 (0.4%)	0.916
Apixaban	62 (3.7%)	24 (3.0%)	38 (4.4%)	0.142
Rivaroxaban	68 (4.1%)	20 (2.5%)	48 (5.6%)	0.002
Edoxaban	27 (1.6%)	7 (0.9%)	20 (2.3%)	0.021
Phenprocoumon	51 (3.1%)	27 (3.4%)	24 (2.8%)	0.461
ACE inhibitors	580 (35.0%)	253 (31.9%)	327 (37.8%)	0.011
Calcium antagonist	355 (21.4%)	160 (20.2%)	195 (22.5%)	0.235
Beta blocker	1,009 (60.8%)	493 (62.1%)	516 (59.7%)	0.310
Antidiabetic drug	261 (15.7%)	104 (13.1%)	157 (18.2%)	0.005
Metformin	201 (12.1%)	78 (9.8%)	123 (14.2%)	0.006
Sulfonyl urea	10 (0.6%)	6 (0.8%)	4 (0.5%)	0.441
Glitazone	0 (0.0%)	0 (0.0%)	0 (0.0%)	–
DPP4 inhibitor	55 (3.3%)	19 (2.4%)	36 (4.2%)	0.044
SGLT2 inhibitor	56 (3.4%)	21 (2.6%)	35 (4.1%)	0.114
GLP1 analogue	23 (1.4%)	11 (1.4%)	12 (1.4%)	0.997
Glinide	0 (0.0%)	0 (0.0%)	0 (0.0%)	–
Any other oral antidiabetic	40 (2.4%)	19 (2.4%)	21 (2.4%)	0.963
Insulin	130 (7.8%)	57 (7.2%)	73 (8.4%)	0.340

#### Differences Between PCSK9i Receivers and Non-Receivers

Patients on PCSK9i were about 2 years younger and had a lower proportion of males. In terms of comorbidities, they had (statistically significantly) more often CAD, but less often PAD, diabetes mellitus, arterial hypertension, and chronic kidney disease. In terms of medications, PCSK9i receivers had less often clopidogrel, NOAC, ACE inhibitors, and oral antidiabetic drugs. Other differences did not reach statistical significance.

#### Lipid Laboratory Values

An overview of lipid values is presented in Table 4. In the total cohort, at baseline median total cholesterol was 164 mg/dl, LDL-C 92 mg/dl, HDL-C 49 mg/dl, non-HDL cholesterol 97 mg/dl, triglycerides 137 mg/dl, and Lp(a) 48 mg/dl.

**Table 4:** Lipid profiles at baseline

Parameter	Total *N* = 1659	PCSK9i receiver, ongoing^a^ *N* = 523	PCSK9i receiver, newly treated* *N* = 271	PCSK9i non-receiver *N* = 865	*P* value
Total cholesterol in mg/dl, median (IQR)	164 (133; 214)	143 (116; 178)	192 (152; 252)	172 (140; 220)	< 0.001
HDL cholesterol in mg/dl, median (IQR)	49 (41; 60)	51 (43; 63)	48 (41; 58)	48 (40; 58)	< 0.001
Non-HDL cholesterol in mg/dl, median (IQR)	97 (68; 156)	77 (50; 102)	169 (109; 217)	118 (86; 174)	< 0.001
Triglycerides in mg/dl, median (IQR)	137 (94; 204)	130 (89; 198)	151 (100; 225)	135 (94; 201)	0.038
Lipoprotein(a) in mg/dl, median (IQR)	48 (10; 122)	44 (8; 96)	58 (8; 118)	58 (12; 125)	0.165
LDL cholesterol in mg/dl, median (IQR)	92 (65; 137)	69 (47; 101)	118 (81; 170)	99 (75; 145)	< 0.001
LDL cholesterol before start of treatment with PCSK9i in mg/dl, median (IQR)	147 (108; 191)	150 (110; 195)	143 (103; 182)		0.058
Calculated untreated LDL-C, mg/dl, median (IQR)	188 (129; 257)	187 (127; 270)	212 (132; 277)	179 (129; 257)	< 0.001
LDL-C goal achievement of < 70 mg/dl, *n* (%)	461 (30.2%)	265 (51.9%)	50 (18.7%)	146 (19.4%)	< 0.001
LDL-C goal achievement of < 55 mg/dl, *n* (%)	258 (16.9%)	181 (35.5%)	24 (9.0%)	53 (7.1%)	< 0.001

To convert the values for lipoprotein(a) to nanomoles per liter, multiply by 2.5. To convert the values for cholesterol to millimoles per liter, multiply by 0.02586. To convert the values for triglycerides to millimoles per liter, multiply by 0.01129. HDL denotes high-density lipoprotein, LDL lowdensity lipoprotein

^a^PCSK9i receiver, ongoing: start of treatment with PCSK9i for more than 30 days before baseline *PCSK9i receiver, newly treated: start of PCSK9i within 30 days before baseline documentation. IQR = interquartile range, SD = standard deviation

The portion of patients with LDL-C < 70 mg/dl was 30.2% and with < 55 mg/dl 16.9%. Distance to goal in 10-mg/dl intervals is shown in Supplementary Fig. 1.

**Fig. 2:**
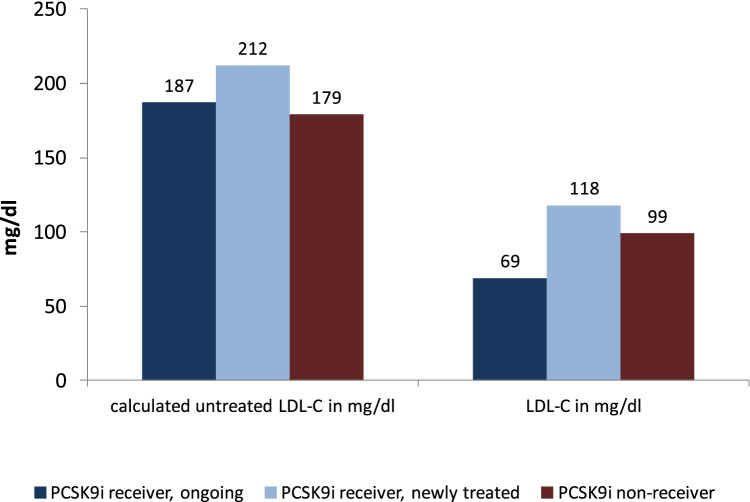
LDL-C values: calculated untreated and measured at baseline. Left: the calculated untreated LDL-C values were higher in patients who were on PCSK9i therapy compared to those newly treated with PCSK9i or not on PCSK9i (median values pre-treated 187 mg/dl [IQR 127;270] vs. newly treated 212 mg/dl [IQR 132;277] versus no PCSK9i 179 mg/dl [129;257]. Right: LDL-C values as measured at study entry (baseline). The most recent available value was used

The median calculated LDL-C value before initiation of any LLT was 187 mg/dl (IQR 127;270) in the group of PCSK9i pretreated patients, 212 mg/dl (IQR 132; 277) in newly treated patients, and 179 mg/dl (IQR 129; 257) in PCSK9i non-receivers (Fig. 2). The distribution of LDL-C values is shown in Supplementary Fig. 2.

#### Statin Intolerance

In the breakdown by intensity, statins of low intensity were given in the total population in 4.0%, of moderate intensity in 42.8%, of high intensity in 53.2% (Table 2).

**Fig. 3:**
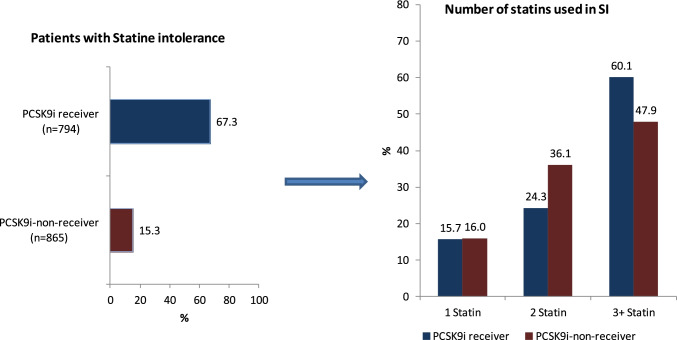
Statin intolerance at inclusion

Among PCSK9i receivers, 534 (67.3%) had statin intolerance (SI) by physician assessment, while among PCSK9i non-receivers, 132 (15.3%) had SI. The majority of patients reported SI for at least 2 statins (Fig. 3). In the 630 patients with available information, 1 statin had been tried in 15.7%, 2 in 26.5%, and 3 or more in 57.8%. Myalgias or myositis combined were reported in 450 PCSK9i receivers (71.4%) (Table 5).

**Table 5:** Statin intolerance and muscle related symptoms at baseline

Parameter	Total *N* = 1659	PCSK9i receiver *N* = 794	PCSK9i non-receiver *N* = 865	*P* value
Statin intolerance	666 (40.1%)	534 (67.3%)	132 (15.3%)	< 0.001
Number of statins tried among patients with statin intolerance^a^				0.022
1 statin	99 (15.7%)	80 (15.7%)	19 (16.0%)	
2 statins	167 (26.5%)	124 (24.3%)	43 (36.1%)	
3 + statins	364 (57.8%)	307 (60.1%)	57 (47.9%)	
Musculature side effects in patients ever treated with statin				< 0.001
Unknown	294 (26.0%)	105 (16.6%)	189 (37.9%)	
Myalgia/myositis/rhabdomyolysis	559 (49.5%)	450 (71.4%)	109 (21.8%)	
None of the above 3 muscular side effects	277 (24.5%)	76 (12.0%)	201 (40.3%)	

Details of the calculation are presented in the methods section

^a^*N* = 36 patients without a report about the number of statins tried (23 PCSK9i receivers, 13 non-receivers)

## Discussion

The present large observational study provides real-world evidence on two groups of patients with dyslipidaemia at very high CV risk, PCSK9i receivers and patients qualifying for but not receiving PCSK9i. As key findings, PCSK9i receivers were characterized by higher baseline LDL-C and a higher portion of statin intolerance compared to those qualified for but not-receiving PCSK9i treatment. PCSK9i receivers versus non-receivers were more likely to have CAD, less likely to have PAD, and less likely to have diabetes. Further, in PCSK9i, receivers on-treatment LDL-C was lower.

Access to PCSK9i is limited by national regulations and many high-risk cardiovascular patients do not receive these therapies [9, 10]. It is of high interest to study whether specific characteristics are more likely associated with individuals receiving PCSK9i or not. A cross-sectional study performed early after the introduction of PSCK9i in the USA showed that at that time these drugs were prescribed appropriately, in line with the summary of product characteristics [12]. In that study, PCSK9i-treated patients had higher rates of cardiovascular comorbidities and physician-determined statin intolerance, had higher LDL-C levels, and received more lines of therapies than non-PCSK9i patients. The PERI-DYS study documents treatment characteristics of patients eligible to receive PCSK9-I in the setting of a different health care system and a different geographic region at a later period in the life cycle of PCSK9i (with physicians having gained more experience with this drug class). PERI-DYS also documents the course and effects of treatment in the long-term. The results will help to identify opportunities to improve patient care. Therefore, PERI-DYS was established as a prospective observational indication registry that will also document the course and effects of treatment in the long-term.

The present cross-sectional analysis of a large sample provides a comprehensive overview on patients with dyslipidaemia at very high cardiovascular risk that fulfill the criteria for treatment with PCSK9i. PCSK9i receivers and those qualifying for but not treated with PCSK9i were eligible for enrolment. Groups differed in some baseline characteristics (e.g., PCSK9i receivers compared to non-receivers were younger and less often males), comorbidities, and also with respect to their lipid profiles: PCSK9i receivers had higher calculated LDL-C values without any LLT therapy, higher LDL-C values before initiation of the PCSK9i therapy (before inclusion into the registry), and higher measured LDL-C values (at inclusion into the registry).

Further, physician-reported statin intolerance was much more common in the two PCSK9i groups compared with the non-PCSK9i group (67.3% versus 15.3%). Patients in the PCSK9i groups received fewer concomitant statins, which is in line with the far higher portion of statin-intolerant patients in this group. Mean on-treatment total cholesterol and LDL-C were considerably lower in patients who were on PCSK9i compared to non-PCSK9i. Interestingly, there were no significant differences observed between patients who were under office-based physician’s care as compared to those under hospital-based care, as well as there were no significant differences based on the HCP´s specialties. The latter suggests that similar decision-making criteria were applied whether to initiate PCSK9i treatment (data not shown).

The potential target population of PCSK9i is large: In a recent published simulation study based on the SWEDEHEART registry with patients with a recent myocardial infarction, the LDL-C goal was achieved in 19.9% of patients if maximum high-intensity statin monotherapy was used, or in another 28.5% with maximum high-intensity statins plus ezetimibe, while 50.7% of patients would still be eligible (and would need) for PCSK9i [11, 13]. These data are consistent with other recent European [14] and German data [15]. The portion of patients actually treated with PCSK9i in Germany is considerably lower than the portion of eligible patients.

In accordance with several other recent reports, LDL-C goal attainment remains a shortfall across all categories of high-risk and very-high-risk patients [16]. Reasons may include caution of physicians to prescribe potent statins in high doses even in very high risk patients [14, 17], in particular to avoid a potentially increased rate of side effects after up titration [18]. Notably, the risk of side effects, in particular “myopathy” under statin therapy in high quality clinical research is very low, which is in stark contrast to the experience during patient care [19].

Published real-world data on PCSK9i is growing while data on use in Germany is still limited to date [20, 21]. To the best of our knowledge, PERI-DYS is the largest prospective observational study on PCSK9i in Germany. The observational studies PEARL [21] and OPTIMIZE [22] that covered, after introduction of alirocumab in Germany, the real-life treatment experience of 619 and 240 patients, respectively. In view of the prescription restriction of the G-BA, similar patient populations were observed. However, regarding specific patient characteristics, there may be inter-study variabilities.

The PERI-DYS data show that the overall patient characteristics of PCSK9i receivers in real world were similar to those in the FOURIER study (mean age 62.5 years, 75.4% males, 80.9% post myocardial infarction, 13.5% PAD, 80.1% hypertension) [7]. However, compared to FOURIER in which all patients were on baseline statins (69.5% high intensity) and few on ezetimibe (5.2%), in PERI-DYS only 52.6% of PCSK9i receivers were on any statin, but 48.2% had ezetimibe. In FOURIER, starting from a baseline value of 92 mg/dl, patients reached a median LDL-C value of 30 mg/dl and maintained it over time. In contrast, in PERI-DYS patients who were already treated with PCSK9i before inclusion, the LDL-C value at inclusion into the registry was 81 mg/dl. Median LDL-C prior to PCSK9i initiation was 159.1 mg/dl (ongoing PCSK9i) and 149.4 mg/dl (newly PCSK9i treatment), which is consistent to other reported real-world data like SAFEHEART registry, in which a median LDL-C of 145 mg/dl prior to PCSK9i was reported [23]. Only 16.9% of patients in PERI-DYS achieved at baseline LDL-C < 55 mg/dl [7], whereas 30.2% of the whole population achieved the 2016 LDL-C treatment goal < 70 mg/dl (51.9% of ongoing PCSK9i, 18.7% of newly-treated at baseline). The fine resolution in the histogram on distance to LDL-C goal (supplement) underlines the higher likelihood for patients to be far from target value in the PCSK9i non-receivers group (with a more pronounced pattern in this direction at baseline).

In the newly treated PCSK9i receivers, we assume that the PCSK9i LDL-C lowering is not yet fully effective at enrolment due to a short period between laboratory measurement and patient enrolment. This result is in line with other reports but varied in comparison to the data from Switzerland (75% of patients < 70 mg/dl) [24] and data from Phar Metrics observations (62.1–69.7% < 70 mg/dl) [25]. Importantly, our study shows that PCSK9i are preferentially used in patients with considerably higher baseline LDL-C compared to the clinical development studies. The fact that eligible patients not on PCSK9i had markedly lower baseline LDL-C strongly supports the assumption that very high LDL-C is one of the main reasons for initiation of PCSK9i in Germany. Since ODYSSEY [26] and FOURIER [7] demonstrated the continuous reduction of ASCVD risk with lower achieved LDL-C—without a lower limit—our data identify an underutilized opportunity for risk reduction with PCSK9i in high-risk patients with moderately elevated baseline LDL-C.

The second important finding relates to the very high number of patients with statin associated symptoms in the PCSK9i group. In fact, two-thirds of the PCSK9i receivers were statin-intolerant. These findings are in line with those from the PEARL observational study in Germany, in which 72.8% of PCSK9i receivers reported complete or partial statin intolerance [21]. Recently published data from an observational study in Switzerland showed a comparable portion of patients with self-reported statin associated muscle syndromes [24]. Also, the German OPTIMIZE study reported a similar proportion of patients with statin intolerance.

The inability to tolerate a moderate or high dose of a statin is an important reason for high baseline LDL-C in these patients. The observed lower on-treatment LDL-C levels confirm the efficacy of PCSK9i in a population were the most important component of background lipid lowering therapy is absent. The data suggest that PCSK9i in Germany are primarily used in patients with very high risk, very high LDL-C and statin intolerance. This finding at the same time identifies large groups that would benefit from PCSK9i according to the guidelines and that would meet the strict requirements for prescription.

In view of the temporal context of the registry study, recruitment started in 2017, i.e., one and a half years after the launch of PCSK9i in 2015 and the introduction of the G-BA regulations and ended in 2021 with new ESC/EAS dyslipidemia guidelines being in place for almost 2 years.

### Limitations

A general limitation of an observational study is the lack of randomization, which would ensure balanced distribution of patients across groups. Patients and physicians who agree to study participation might differ from those who decline it, which may cause a bias toward better treatments, e.g., lower achieved LDL-C concentrations. The exact decision-making process for assigning specific LLT (PCSK9i or not) in individual patients cannot be assessed in this study; however, associations observed between the groups may indicate such reasons. Results of the LDL-C calculation may well be in the right ballpark overall, however in reality a substantial variation in achieved LLT effects can be expected across patients.

The LDL-C calculation was based upon the well-established effects size of LDL-C reduction of the respective LLT in randomized clinical studies. However, it still carries potential variation bias due to individual patient response to LLT drugs, uncertainty in terms of LLT medication compliance, and variation in the timing of laboratory assessments.

In conclusion, the PERI-DYS data provide robust and up-to-date evidence showing that patients treated with PCSK9i are characterized by higher baseline LDL-C and a higher rate of statin intolerance compared to those qualifying for but not treated with PCSK9i. Despite the limitations of an observational study, the baseline characteristics indicates that the patient selection for this registry was in line with the characteristics laid out by national reimbursement criteria in Germany. The selection of PCSK9i in a group with mainly statin intolerance is in line with previous data demonstrating that PCSK9i may offer a very relevant contribution to LDL-C lowering once the important pillar of statins cannot be tolerated in the required intensity [27]. Importantly, the ongoing follow-up of the PERI-DYS registry will determine the prognostic importance of these findings.

### Supplementary Information

Below is the link to the electronic supplementary material.

Supplementary file1 (DOCX 137 KB)

## Data Availability

Qualified researchers may request data from Amgen clinical studies. Complete data are available at the following: https://wwwext.amgen.com/science/clinical-trials/clinical-data-transparency-practices/.
