# Supplementary material for: Novel Insights into the Management of Patients with Very High Cardiovascular Risk Eligible for PCSK9 Inhibitor Treatment: Baseline Findings from the PERI-DYS Study
Source: Cardiovasc Drugs Ther. 2022 Sep 30;38(1):119–29. doi: 10.1007/s10557-022-07386-0 (PMC10876819; doi:10.1007/s10557-022-07386-0)
Supplement: Supplementary file 1 — Supplementary file1 (DOCX 137 KB) [file 10557_2022_7386_MOESM1_ESM.docx]

**Supplementary Table 1. Variables at inclusion and follow-up**

| **Variables** | **Inclusion documentation** | **Follow-up documentation** |
| --- | --- | --- |
| Check of eligibility criteria, informed consent | ✓ |  |
| Patient demographics | ✓ |  |
| Cardiac comorbidities and risk factors ^1^ | ✓ |  |
| Lipid laboratory values, if available  (LDL-C, HDL-C, TG, TC, Lp(a)) | ✓ | ✓ |
| Lipid lowering therapy  (drug treatment, lipid apheresis) ^2^ | ✓ past and current | ✓ |
| Non-LLT cardiac medication ^3^ | ✓ past and current | ✓ |
| MACE events ^4^ including cardiovascular interventions | ✓ past and current | ✓ |
| Hospitalisation and rehabilitation |  | ✓ |
| Adverse Drug Reaction ^5^ |  | ✓ |
| QoL by EQ-5D | ✓ | ✓ |
| ^1^ Diabetes mellitus, arterial hypertension, heart insufficiency, respiratory insufficiency, coronary artery disease (and last acute coronary syndrome event), cerebrovascular event, peripheral arterial occlusive disease, atrial fibrillation/flutter, renal insufficiency; prior and current smoking.  ^2^ PCSK9i, statins, ezetimibe, nicotinic acid, fibrates, cholestagel, omega-3 fatty acids. Any combinations of the mentioned agents will be documented, too.  ^3^ Beta blockers, ACE inhibitors, ATII antagonists, nitrates, ASA, diuretics, insulin, other antidiabetic drugs  ^4^ Death (cardiovascular, non-cardiovascular), acute coronary syndrome (STEMI, NSTE-ACS, UA), cerebrovascular event.  ^5^ ADR to be reported within 1 business day of physician awareness. | | |

**Supplementary Figure 1. Distance to LDL-C goal 70 mg/dl**

Columns show 10 mg/dl intervals, e.g., the first column on the left indicates that 40.3% of PCSK9i receivers are at 70mg/dl or below, 7.9% are 1 - 10 mg/dl above the goal value, 7.9% are 11 - 19 mg/dl above the goal.

**Supplementary Figure 2. Distribution of calculated LDL-C values prior to initiation of lipid-lowering treatment**

Details of the calculation are presented in the methods section.
